# Mass Transfer and Optical Properties of Active PET/PP Food-Grade Films Impregnated with Olive Leaf Extract

**DOI:** 10.3390/polym14010084

**Published:** 2021-12-27

**Authors:** Cristina Cejudo Bastante, Marlene J. Cran, Lourdes Casas Cardoso, Casimiro Mantell Serrano, Stephen W. Bigger

**Affiliations:** 1 Chemical Engineering and Food Technology Department, Wine and Agrifood Research Institute (IVAGRO), University of Cadiz, 11519 Cadiz, Spain; lourdes.casas@uca.es (L.C.C.); casimiro.mantell@uca.es (C.M.S.); 2 Institute for Sustainable Industries and Liveable Cities, Victoria University, P.O. Box 14428, Melbourne, VIC 8001, Australia; marlene.cran@vu.edu.au (M.J.C.); stephen.bigger@vu.edu.au (S.W.B.)

**Keywords:** kinetic migration, CIELAB parameters, supercritical impregnation, olive leaf extract, UV barrier, impregnation mode

## Abstract

A supercritical solvent impregnation (SSI) technique was employed to incorporate, by batch- and semicontinuous-modes, bioactive olive leaf extract (OLE) into a food-grade multilayer polyethylene terephthalate/polypropylene (PET/PP) film for active food packaging applications. The inclusion of OLE in the polymer surfaces significantly modified the colour properties of the film. A correlation of 87.06% between the CIELAB colour parameters and the amount of the OLE impregnated in the film was obtained which suggests that colour determination can be used as a rapid, non-destructive technique to estimate the OLE loading in the impregnated matrices. The UV barrier and water permeability properties of the films were not significantly modified by the incorporation of OLE. The migration of OLE into a 50% (*v*/*v*) ethanol food simulant demonstrated faster release of OLE from the PP surface than from the PET surface which may be due to the different interactions between OLE and each polymer.

## 1. Introduction

Given the perishable nature of fresh produce, packaging is an investment necessary for producers to protect and preserve products from post-harvest through to sales and distribution. However, the growing need for food preservation has resulted in the excessive use by the food industry of synthetically derived additives. Over recent years, the focus has shifted towards finding alternatives to the direct addition of chemical preservatives and the field of active packaging materials has subsequently gained attention. This has resulted in improvements in food contact packaging materials by the incorporation of natural additives that can interact with the food and surrounding environment to minimise food spoilage [1,2,3].

In general, active packaging describes a system in which the materials actively participate in food preservation by different mechanisms, such as delaying lipid oxidation, inhibiting microbial growth, inhibiting absorption of moisture or vapour exchange, among others [4]. This type of packaging is designed to incorporate active components into an inert polymer matrix to either absorb undesired compounds, such as gases or off-flavours, or to be released into the package headspace [5]. These compounds are generally chemical substances and are commonly agents that are naturally derived from plants or agricultural by-products. Traditionally, volatile compounds derived from essential oils and extracts with antibacterial and antioxidant capacities have been used because of their propensity to be easily released into the headspace of the packaging [6,7,8]. However, there is a wide range of non-volatile compounds, such as phenolic-rich extracts, that can also be used for such purposes, with many having potent biological activity that can assist in preventing or minimizing oxidation and food spoilage [3,9,10,11,12,13,14].

Supercritical solvent impregnation (SSI) is a technique employed to impregnate different matrices with both volatile and non-volatile compounds to impart antioxidant, antibacterial or other active properties [15,16,17,18]. Supercritical CO_2_ (scCO_2_) has the dual ability to solubilise the active compound, with or without the addition of a co-solvent, and to act as a plasticizer of the polymer matrix by opening its chains and favouring the subsequent solute-matrix reaction [18,19]. This technique takes advantage of the low temperatures required for the impregnation (ca. 35–55 °C) and offers the benefit of activating the polymer film after it is formed by conventional thermal processing. Thus, the functionalization assisted by scCO_2_ of already formed polymers protects the active compounds from the high temperatures (ca. 170 °C) usually required for the incorporation of the additives during film extrusion.

The SSI technique can be performed in batch or semi-continuous mode using one or two vessels [20,21,22]. Batch mode (BM) involves a closed system where the amounts of CO_2_ (solvent) and extract are fixed, whereas semi-continuous mode (SM) allows a constant renewal of either or both of the solvent and extract, which can possibly improve impregnation yields. Few reports in the literature compare the properties of films impregnated using these different processes, with the majority of studies generally focused on the impregnation loadings obtained. For example, Milovanovic, Hollermann, Errenst, Pajnik, Frerich, Kroll, Rezwan and Ivanovic [20] evaluated the impregnation loading and thermogravimetric properties of poly(lactic acid)/polycaprolactone films impregnated with thymol using BM and those impregnated by an integrated supercritical extraction-impregnation-SSI process using thyme with CO_2_ recirculation. In another study, García-Casas, et al. [23] compared the loading of quercetin into silica by different impregnation modes but did not report the physical properties of the material before and after impregnation.

In the case of food packaging films subjected to the SSI process, the analysis of barrier properties is particularly important due to the high pressure conditions that are used, as well as the CO_2_ sorption on the amorphous phase of the polymer which could render the material unsuitable for packaging purposes [24]. In addition to the processing conditions, the inclusion of a foreign substance, such as a natural extract, into the polymeric formulation may also result in adverse effects on key film properties. The characteristics of the film, including colour, UV barrier properties, and water permeability are parameters commonly measured to evaluate the suitability of a material for food packaging [4,13,25]. In the case of active packaging, another important parameter is the release of the active substance from the film matrix, which is directly related to the preservative efficacy of the film. The migration of an active compound depends on its affinity with the polymer matrix, the food or simulant composition, and a range of environmental factors [12,26,27,28].

In our previous work, polyethylene terephthalate (PET)/polypropylene (PP) films subjected to supercritical impregnation of olive leaf extract (OLE) by the aforementioned BM and SM impregnation modes were characterized in terms of mechanical properties and antioxidant activity [29]. The present work aims to further characterise these films in terms of their barrier parameters, colour, water permeability and UV barrier properties, as well as study the mass transfer of OLE from the films under optimum impregnation conditions.

## 2. Materials and Methods

### 2.1. Chemical Reagents and Raw Materials

Carbon dioxide (99.99%) was purchased from Abello-Linde S.A. (Barcelona, Spain). Technopack Univel S.r.L. (Mortara, Italy) provided the film used in the experiments which was comprised of PET and PP layers of 12 and 50 μm thickness, respectively.

The films were impregnated with the extract from *Olea europea* leaves that were obtained from the region of Jaén (Andalucía, Spain). The extract was obtained by treating the leaves in accordance with a method described by Cejudo Bastante, et al. [30]. Briefly, an enhanced solvent extraction was carried out with 200 g of olive leaf in a 1 L vessel at 120 bar and 80 °C using CO_2_ solvent modified with ethanol (1:1).

### 2.2. Supercritical Impregnation Procedures

The film impregnation procedures were performed as previously described [31] and the different modes were performed as follows:

1. In BM, the impregnation was carried out with the vessel outlet closed to maintain a fixed amount of scCO_2_ inside the vessel. Once the pressure conditions were achieved, the impregnation commenced and the atmosphere inside the vessel was homogenized with an upper agitation system stirring at 40 rpm.
2. In SM, the scCO_2_ was renewed in the system at a flow of 2 g min^−1^ to maintain the pressure inside the vessel. In this case, the scCO_2_ stream efficiently agitates the agents inside the vessel, and thus an agitation system was not required.

For both impregnation modes, the conditions were: 100 and 400 bar, 35 and 55 °C, and the depressurization rate was 100 bar min^−1^ after 1 h of impregnation.

### 2.3. Optical Properties

The surface colour of the films was evaluated using a Chroma meter (Konica Minolta, CR-400, Tokyo, Japan). The CIELAB coordinates, L* for luminosity (black (0) to white (100)), a* (green (−) to red (+)) and b* (blue (−) to yellow (+)), were measured using a standard D65 illuminant and were acquired from the reflection spectra of the samples placed on a white calibration plate (L* = 97.39, a* = 0.03 and b* = 1.77). The average of nine readings from each film sample was measured to calculate the average colour values.

The UV barrier properties were analyzed in duplicate using a UV-visible spectrophotometer (UV-1800, Shimadzu Corp, Kyoto, Japan) following the method described by Kuorwel, et al. [31]. A rectangular piece of film (0.5 × 1.5 cm) was placed in the spectrophotometer cell and, using air as a reference, the opacity (*Op*) was calculated by measuring the absorbance at a wavelength of 500 nm and the transparency (*Tr)* was calculated using the percentage transmittance of light at 600 nm as a function of the film thickness (*x*) [32]:

(1) Op=Abs(500)/x

(2) $$Tr=Log(10^{-Abs\;600}\times 100)/x$$

### 2.4. Water Vapour Permeability

Water vapour permeability (WVP) was determined at two temperatures using the method described in ASTM E96-95. Circular film samples of 1 cm diameter were mounted on the top of 10 mL sample bottles with 5 mL of water and sealed tightly to prevent leakage of water vapour. Samples were then placed in a desiccator stored at 20 °C and in an air-circulating oven set at a temperature of 35 °C. The flasks were weighed periodically for up to 20 days and the loss of water was calculated as the %WVP as follows:

(3) $$\mathrm{WVP}=\left(W_{1}-W_{2}\right)/W_{1\;}\times 100$$

where *W*_1_ and *W*_2_ are the initial and final weights of the flasks, respectively.

### 2.5. Mass Transfer of OLE into Food Simulant

The release of active compounds into a food simulant is commonly used to predict the migration profile in real food systems [26,33,34]. In the present study, a 50% (*v*/*v*) ethanol/water food simulant was used to mimic foods with high lipid content. Moreover, the presence of ethanol has been observed to favour the migration of OLE compounds from PET/PP films [35]. The release profile of OLE from both the PET and PP surfaces was studied separately for the PET/PP films by sealing tubes containing 5 mL of food simulant with 3 cm diameter pieces of film sample (ca. 100 mg). The tubes were placed upside down to allow contact between the simulant and the film to facilitate migration of OLE and at 10-min intervals, ca. 3 mL of the simulant was placed into a quartz cuvette and the absorbance measured at 667 nm using a Horiba Aqualog^TM^ spectrofluorometer (HORIBA, Jobin Yvon Technology, Kyoto, Japan). The sample was returned to the tube to maintain a constant volume in the tube and the tube was then placed back in contact with the film until the next measurement. Solutions were agitated prior to sampling to homogenise the samples and the absorbance of the simulant was recorded until the determination was stable. Three replicates were performed for each side of the film so that both the PP and PET surfaces were in contact with the simulant. Considering that the simulant was not stirred during the assay, the transfer mechanism assumed for this system was lineal diffusion.

The concentration of OLE (*C_OLE_*) in the simulant was determined using a calibration curve of OLE (5–100 ppm) previously derived [30]:

(4) $$Abs=18.957\times C_{OLE}+83.591\;\;\;\;\left(R^{2}=0.9855\right)$$

The release of additives can often be described by simple first order kinetics [26,36]:

(5) $$m_{t}=m_{\infty }\left(1-e^{-k_{1}t}\right)$$

where *m_t_* is total amount of diffusing substance at a time *t*, *m_∞_* is the equilibrium amount of the compound that migrates at infinite time, and *k*_1_ is the first-order rate constant. Differentiating both sides of this equation with respect to time gives:

(6) $$\frac{dm_{t}}{dt}=m_{\infty }k_{1}\left(e^{-k_{1}t}\right)$$

The initial release rate is an important consideration in the characterization of active films and is directly related to the effect of the active agent on the food in the initial stages of contact. The solution to Equation (6) when *t* = 0 provides an estimation of the initial release rate ($v_{0}$) of the OLE into the food simulant [37]:

(7) $$v_{0}=m_{\infty }k_{1}$$

In some cases, the value of *m_∞_* may not be known and this is common when the migration of active substances is very slow. It may therefore be necessary to estimate the value of *m_∞_* and this can be achieved using the first-order kinetic model by rearranging Equation (5):

(8) $$\ln\left(1-\frac{mt}{m\infty }\right)=-k1t$$

A plot of ln(1 − *m*_t_/*m_∞_*) *versus* time is a straight line with slope -*k*_1_. Using an iterative least squares approach, the linearity of this fit can be optimized by estimating the value of *m_∞_* which can be used in subsequent calculations.

In addition to the first-order kinetic approach, the migration of OLE into the food simulant was determined using the mathematical model based on Fick’s second law:

(9) $$\frac{\partial C_{p}}{\partial t}=D\frac{\partial^{2}C_{p}}{\partial x^{2}}$$

where *C_p_* is the concentration of the migrant in the polymer at time, *t*, and position, *x*, and *D* is the diffusion coefficient.

In the case of mass transfer of a solute impregnated in a film, the value of *D* can be related to the capability of the solute to gain access to the food and to thereby develop its effect. High values of this coefficient generate a high presence of the solute in the food, but the effect over time may decrease. In the case of a laminate film in an infinite medium Equation (5) may be solved to determine the value of *D*. One common solution to this equation is presented by Crank [37] who described the migration of small molecules from a polymeric film into an infinite volume of solvent. In this derivation, the value of *D* is related to the total amount of diffusing substance (*m_t_*) at a fixed time interval (*t*), the equilibrium amount of the compound that migrates at infinite time (*m_∞_*), and the polymer thickness (*l*), in accordance with the following equation:

(10) $$\frac{mt}{m\infty }=1-\frac{8}{\pi^{2}}\overset{\infty }{\underset{n=0}{\sum }}\frac{1}{{\left(2n+1\right)}^{2}}\exp\left[\frac{-D{\left(2n+1\right)}^{2}\pi^{2}t}{l^{2}}\right]$$

In order to calculate the optimum value of *D*, the experimental data can be fitted to Equation (10) using an iterative technique [36] that minimizes the sum of the squares of the residuals between the predicted and actual values.

### 2.6. Statistical Analysis

A non-supervised chemometric tool, namely a hierarchical cluster analysis, was used to evaluate the colour parameters of the samples. In addition, a multiple variable analysis was performed (*p* < 0.05) to determine any correlation among colour parameters and impregnation loading of films. Statistical differences among samples were determined using analysis of variance (ANOVA, *p* < 0.05) and Fisher’s least significant difference. All statistical analyses were performed using the statistical package Statgraphics Centurion XVI 16.1.

## 3. Results and Discussion

### 3.1. Optical Properties of Films

Colour characteristics and the UV barrier (transparency and opacity) are key properties in food packaging materials that can facilitate observations of food products by consumers and can influence the acceptability of produce [11,38]. Many of the degradation reactions of food are light dependent, being more pronounced in the UV region. To avoid these reactions, the inclusion of UV blockers into the package to absorb some wavelengths has been utilized in film formulations in order to increase the shelf-life of different products [14,39]. In the present study, the change of these properties following impregnation with OLE under the different conditions of pressure, temperature, and mode of impregnation were evaluated.

#### 3.1.1. Colour Properties

**Figure 1:**
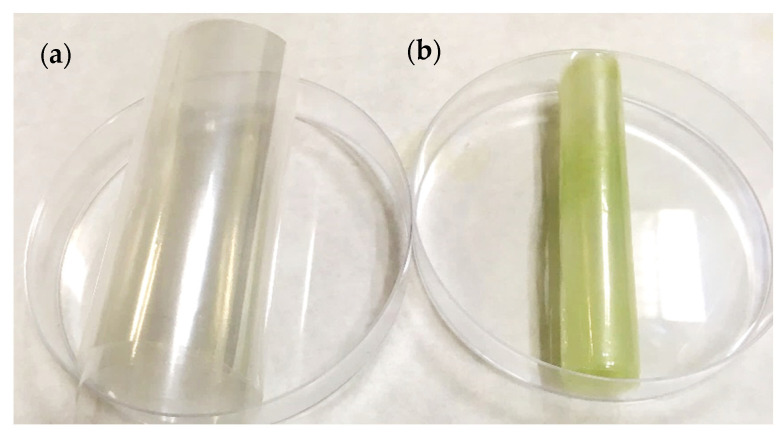
Appearance of the PET/PP films (**a**) before and (**b**) after OLE impregnation (sample corresponding to the impregnation at 400 bar and 35 °C by batch mode).

As shown in Figure 1, the colour of the PET/PP film substantially changed following impregnation with OLE, thus the chromatic characteristics of the films were evaluated.

As shown in Table 1, the impregnation process caused significant changes to the film colour and subsequent CIELAB parameters compared to the neat PET/PP film which was transparent and almost colourless. The lightness (L*) values were very similar to the control although some statistical differences were found when OLE was incorporated (*p* < 0.05) where L* values decreased. This behavior was also observed by Wrona, et al. [40] who incorporated green tea extract and poly(lactic acid) (PLA) nanoparticles into HPMC films and by Wang and Rhim [41] upon the incorporation of grapefruit seed extract into low-density polyethylene (LDPE) and PLA films. In the present study, the samples that showed a statistically different value from the control film were BM at 400 bar/35 °C, BM at 100 bar/55 °C, and SM at 100 bar/35 °C, with these systems generally having a high degree of OLE impregnation. With respect to the a* values, all samples were different to the control, which confirms that the impregnation of OLE significantly imparted a green colouration to varying degrees depending on the conditions. In the case of the b* values, the samples that were significantly different to the control were those with a concurrent greater level of impregnation (i.e., SM at 100 bar/35 °C, and BM at 400 bar/35 °C). Lower L* values combined with higher b* values were indicative of a yellowish film appearance which became more apparent at higher OLE loadings [42]. With regard to the overall colour change (Δ*E*) in samples after treatment, only the sample impregnated at 400 bar/35 °C using SM was statistically similar to the control. Therefore, the OLE impregnation process was confirmed to significantly contribute to the modification of the resulting film colour properties.

Supercritical impregnation technologies have been recently used for the dyeing of polymers and fabrics since they offer more environmentally friendly techniques in contrast to conventional dyeing processes [43]. It is possible to correlate the colour uptake with the impregnation yield and, in one example, Varga, et al. [44] related the colour of polycarbonate to the dye uptake under different pressure, temperature and time conditions. The colour intensity and fastness properties have also been correlated with a bacteria reduction of 75–93% following the impregnation of dyes in polyester fabrics containing chitosan derivatives by supercritical techniques [45]. In another example, Solovieva, et al. determined the molecular form of photochromic spirooxazine dyes incorporated into different polymers based on the variation in colour [46].

**Figure 2:**
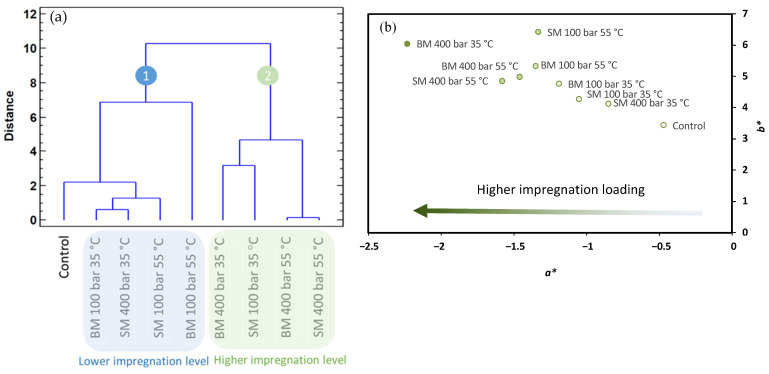
Dendrogram obtained from the HCA of control film and impregnated film considering the CIELAB parameters (**a**); CIELAB coordinates in (a*b*)-plane for control and impregnated films (**b**).

In order to verify whether there is any correlation between the CIELAB coordinates (L*, a*and b*) and the impregnation parameters, data in the present study were submitted to a hierarchical cluster analysis (HCA) where samples were grouped according to the squared Euclidean distance and Ward’s method. The dendrogram resulting from the HCA is shown in Figure 2a.

The results indicate that the operational parameters of pressure, temperature and impregnation mode do not influence the CIELAB parameters. However, the organization of the samples on the HCA is related to the impregnation loading under each condition. The OLE loading obtained in these films was reported in a previous study using an antioxidant assay as an indirect quantification method [29] and these results are summarized in Table 1. The HCA classified the samples into two general groups: (i) the untreated film (control) and the samples with a low level of impregnation (≤1 mg g^−1^ OLE film), and (ii) the samples with a higher level of impregnation (≥1.8 mg g^−1^ OLE film). The sample subjected to BM at 400 bar and 55 °C, that initially showed a higher antioxidant activity, was classified in the first group. When the data are plotted in the CIELAB space (Figure 2b), regardless of the similarities in the values, the organization of the samples in relation to the OLE loading is again evidenced with particular regard to the a* parameter that corresponds to the green colour imparted by OLE to the impregnated film.

In order to confirm a correlation between CIELAB parameters and impregnation loading, a multivariate analysis (*p* < 0.05) was performed. This analysis showed that L*, a* and b* parameters were significantly correlated with the impregnation loading with strong negative correlations in the case of L* and a* (correlation coefficients −0.7237 and −0.8975, respectively), and a strong positive correlation with b* (correlation coefficient 0.8238). A multiple regression analysis fitted the results to a multiple linear model (*p*-value = 0.0117) with the following equation describing the impregnation loading (IL) with the L*, a* and b* values:

(11) $$IL=90.81-0.96L^{*}-1.16a^{*}-0.01b^{*}$$

The *R*^2^ statistic indicates that this model as fitted explains 87.06% of the variability in the impregnation loading.

The quantification of compounds loaded by supercritical impregnation in different matrices including films is generally difficult to analyze due to the low impregnation yields obtained. Some authors have gravimetrically determined the impregnation loading of polymers by weighing film samples before and after processing [5,47,48]. However, this technique is not suitable when low levels of impregnation are obtained, so others have determined the impregnation loading through release studies [15,49]. Other authors have used various indirect techniques to quantify the impregnation loading, including the use of an antioxidant assay or indicators of the bioactivity of the resulting matrices [10,16]. Analytical methods, such as liquid or gas chromatography, have been used, but these require extraction of the impregnated agents from the matrix and complete extraction is not always obtained [50,51]. Nevertheless, to the best of our knowledge, there are currently no literature reports correlating the supercritical impregnation loading of an active agent and the CIELAB parameters of the resulting films. The change of colour in the impregnated matrices compared to the untreated films is clear evidence of the success of OLE impregnation in the present study. The analysis of the CIELAB parameters can therefore be proposed as a non-destructive, fast and efficient tool to predict the loading of an impregnated PET/PP film with OLE, or indeed other films impregnated with coloured pigments or active agents.

#### 3.1.2. UV Barrier Properties

To determine if the incorporation of OLE into treated films increases their UV barrier properties, the transparency and opacity were determined, as shown in Table 2. In general, samples impregnated using SM were comparable to the control film whereas those impregnated using BM showed more variability. However, a univariate analysis of variance (Fisher test and ANOVA, *p* < 0.05) revealed that there was no statistical influence of the operational parameters on the UV barrier properties. All samples were classified within the same group as the control except for the sample impregnated using BM at 400 bar/35 ºC which is the condition that produced films with the highest impregnation loading. This sample showed statistically lower transparency and higher opacity which may be due to the light scattering and/or absorption properties of the OLE compounds which have been observed in similar studies [52].

The consumer acceptability of any packaging film will be highly dependent on the type of food being packaged [11,12,13]. For example, films that are coloured with green pigments, such as OLE, may be more suited to packaging green produce, such as lettuce or other vegetables.

Chalco-Sandoval and co-authors [38] reported that the processing conditions of multilayer films prepared by electrospinning influenced the transparency of the resulting films. In the present study, however, the impregnation conditions did not significantly influence the UV barrier properties with the exception of the case of the highest impregnation loading at 400 bar and 35 °C using BM. Similar results have been observed in the case of LDPE and PLA films loaded with grape seed extract which did not substantially affect the transparency of the films [41], and in the case of chitosan films containing anthocyanin which showed significantly higher opacity when the loading was >3% [53]. Active films obtained under the conditions of maximum impregnation in the present study therefore have the potential to improve the photodegradative protection of packaged food exposed to UV light.

### 3.2. Film Water Vapour Permeability

Analysis of water vapour permeability (WVP) is fundamental to the development of food packaging films in order to evaluate the propensity for water to permeate through the film. The WVP is dependent on several factors, including the packaging environment, amount and distribution of the amorphous region of the polymer (where gas and vapour exchange take place), and film orientation, among others [39]. Greater permeation usually occurs in the hydrophilic parts of the film compared with the hydrophobic regions [54] and therefore surfactants are usually added to hydrophilic materials, such as those made from carbohydrates and proteins, to control the WVP. Excessive transpiration of water vapour through the film into the package headspace can condense and subsequently increase the risk of mold and bacterial growth. Conversely, inadequate transport of water can result in a dry atmosphere which can cause desiccation of some foods.

**Figure 3:**
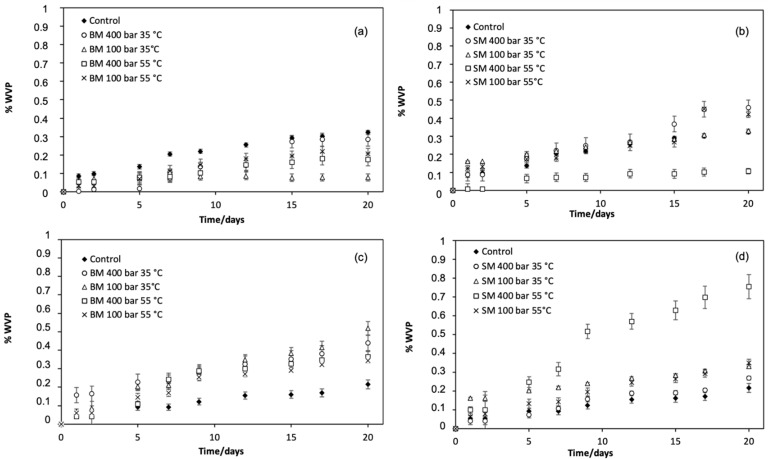
Percentage WVP of untreated PET/PP film (control) and OLE-impregnated films at (**a**,**b**) 20 °C and (**c**,**d**) 35 °C.

In the present study, changes in the %WVP of the film samples over 20 days at 20 and 35 °C are shown in Figure 3. In the case of experiments performed at 20 °C (Figure 3a,b), samples impregnated using BM showed lower %WVP than the untreated control film, whereas those impregnated using SM showed trends in %WVP values similar to the control, with the exception of the sample impregnated at 400 bar/55 °C which showed lower values. This suggests that, for samples with lower WVP, the microstructures of the film may have compacted slightly, thus increasing the resistance to water vapour transport after the impregnation process [53]. When the experiment was performed at 35 °C (Figure 3c,d), differences in the %WVP of the impregnated films compared to the control were more evident. As reported by Sangaj and Malshe, the solubility of water in polymers increases when temperatures increase, thus favouring its permeation [55]. However, increasing the temperature did not seem to influence the WVP of the control film to the same extent as was observed for the treated films, showing similar values in both experiments at 20 °C and 35 °C. For the impregnated films, the effect of the treatment was more apparent and samples showed %WVP values higher than the untreated control. Similar to the case of samples tested at 20 °C, samples impregnated at 400 bar/55 °C using SM produced the highest %WVP value at 35 °C.

Literature reports describe variations in the WVP of films obtained by casting as a result of the presence of loaded active compounds. For example, Siripatrawan and Vitchayakitti [56] reported the reduction of WVP of chitosan films loaded with propolis at concentrations ranging from 0.2 to 20% *w*/*w*. Sanchez-Garcia and Lagaron [57] reported a similar reduction in the WVP of PLA, polyhydroxybutyrate-co-valerate, and polycaprolactone, when a yellow clay was incorporated in their composition. In the case of protein films, Benbettaïeb, et al. [58] reported increases in %WVP when phenolic compounds were added in the range of 40 to 60 mg g^−1^ of film due to the increase in -OH groups from phenolic compounds in the polymeric structure. In the present study, in general, the low level of impregnation did not cause a significant variation in the permeability of the films. Overall, after 20 days of testing, the maximum %WVP did not exceed 0.7% with most samples attaining a maximum <0.5%, so it was concluded that the treatment of the films did not significantly change the water barrier properties.

### 3.3. Evaluation of OLE Migration

The migration of active agents from a polymer matrix is important in characterizing the efficacy of the active agent towards the target function. In the case of multilayer films, where the active agent is present in each surface, the migration from the surface of each side of the film can be measured separately. In the present study, evaluation of the migration of OLE from both the PET and PP surfaces from films obtained at the highest impregnation loadings was studied to observe any differences between the polymer composition and the applied conditions. To achieve this evaluation, the following assumptions were made:

- The OLE compounds were homogeneously dispersed within the matrix.
- The *D* coefficient is constant, considering that the impregnation loading is low.
- The migration occurs in one direction, since only one surface is in contact with the simulant.

**Figure 4:**
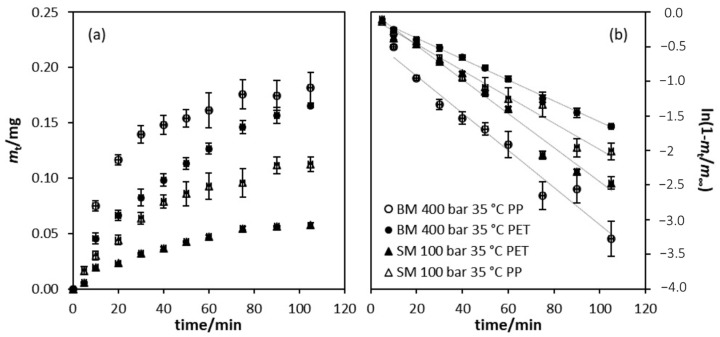
Solute release into the 50% *v/v* ethanol in water food simulant (**a**) and first order kinetics plot of solute release (**b**).

The amount of OLE released from selected films into the food simulant versus time is presented in Figure 4a. It is evident from this plot that films impregnated at 400 bar using BM released more OLE in the first 10 min and that more OLE was released from the PP side of the film. In the case of films impregnated at 100 bar using SM, a similar release profile was observed with the PP surface releasing more OLE into the simulant than the PET surface. Under isothermal conditions, there is generally higher CO_2_ sorption and swelling in polymers which may result in a reorganization of the chains to a more disorganized structure after depressurization. In a previous study [20], the crystallinity of the same laminate film used in the current study was shown to decrease when the film was submitted to high pressures, and was more apparent in the case of the PP surface than the PET surface. Moreover, higher swelling promotes greater penetration of the solute in the bulk of the film [59] which could potentially result in a higher release from the PP surface. The greater mass released from the film impregnated at 400 bar is also consistent with a higher level of OLE impregnation under these conditions.

Theoretically, the total amount of an active agent will only be released after an infinite time and only into an infinite volume of food simulant in the experiment. In reality, for a finite volume of simulant, a distribution equilibrium will be attained, and this is taken as the “infinity” value. Moreover, the amount of an active agent that can migrate from a matrix at any time depends on many factors and will generally be lower than the total amount added. In order to estimate the value of *m*_∞_, plots of ln(1 − *m*_t_/*m*_∞_) versus time for each of the four film samples were prepared and are shown in Figure 4b. In each case, the value of *m*_∞_ was optimized to obtain the best linear fit to this equation. The linearity of these plots, as shown by the high *R*^2^ values (Table 3), confirms the applicability of the overall first-order kinetic analysis to the systems according to Equation (8). This also enables a convenient estimation of the initial rate of release of the AM agent from the derived kinetic parameters in addition to the estimation of the value of *m*_∞_.

**Figure 5:**
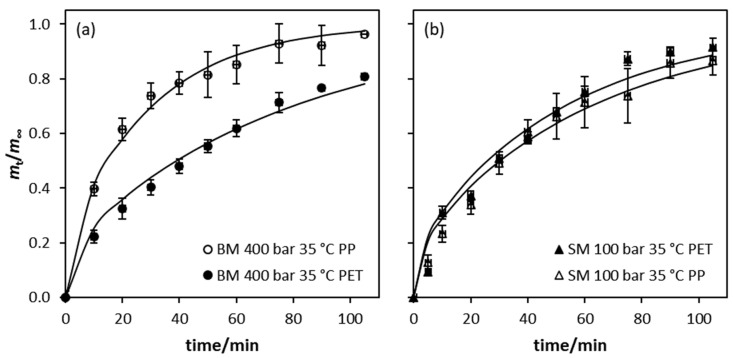
OLE diffusion of samples impregnated at 400 bar 35 °C using BM (**a**) and 100 bar 35 °C using SM (**b**) according to Fick’s law.

Based on the optimized *m*_∞_ values obtained, the data were analyzed by optimizing the values of *D* in accordance with Equation (10). Figure 5 shows the diffusion plots of the OLE migration from the surface of different layers of the films under the two impregnation conditions where the solid lines in the figures represent the fit to Equation (10). For the samples obtained at 400 bar 35 °C using BM (Figure 5a), the migration from the PP surface appeared to approach equilibrium at the end of the test whereas only ca. 80% of the OLE was released from the PET side of the film. In the case of the films obtained at 100 bar 35 °C using SM (Figure 5b), the migration profiles were similar and equilibrium was not reached at the end of the experiment.

As shown in Table 4, the initial release rate of OLE was higher from the PP surface than the corresponding PET surface under both impregnation conditions. In addition, the initial release rate was higher from films prepared under the BM conditions at 400 bar and 35 °C and is in accordance with a higher impregnation loading of OLE. The values of *m_∞_* also varied with impregnation mode and pressure with the lowest value of *m*_∞_ obtained for the PET film surface under SM conditions at 100 bar. The percentage release at the end of the experiment confirms the suggestion that the release did not reach equilibrium for any of the films. The values of *D* obtained using Equation (10) by fitting experimental data to Fick’s second law are generally in agreement with those reported in the literature for similar polymer matrices [60]. Moreover, the correlations between the actual data and the data obtained using the model were >99% in all cases. In general, it is evident that an increased pressure during the impregnation process resulted in a higher impregnation loading which is in agreement with the impregnation of thymol in LLDPE films [61]. In a previous study, a possible chemical interaction between the PET surface and OLE was revealed via Fourier-transform infrared (FTIR) analysis [29]. A reduction of the carbonyl peak suggested the existence of hydrogen bonding between PET and OLE compounds which was not observed between PP and OLE. In the case of the latter, physical retention of OLE was suggested since the methyl groups of PP lack the capacity to interact with the range of substances present in OLE [29]. These observations are consistent with the migration of OLE observed in the present study where the migration of OLE from the PP surface was faster than that from the corresponding PET surface. Similar results have been reported for the release of supercritical impregnated thymol from LDPE and LDPE modified with montmorillonite clay [62]. The neat LDPE polymers exhibited a quick release of thymol reaching a steady state within 2 h which was similar to that observed for the release of OLE from the PP surface in the present study. In the case of LDPE containing montmorillonite, the clay hydroxyl groups formed hydrogen bonds with the thymol, resulting in a slower release, equilibrium attainment after 11 h, and a 166-fold decrease in the diffusion coefficient. Similar results have been reported for the release of thymol from PLA films containing nanofibers with a decrease in the diffusion coefficient from 2.5 × 10^−13^ to 5.5 × 10^−14^ m^2^ s^−1^ due to the interaction between the thymol and the nanofibers [51].

**Figure 6:**
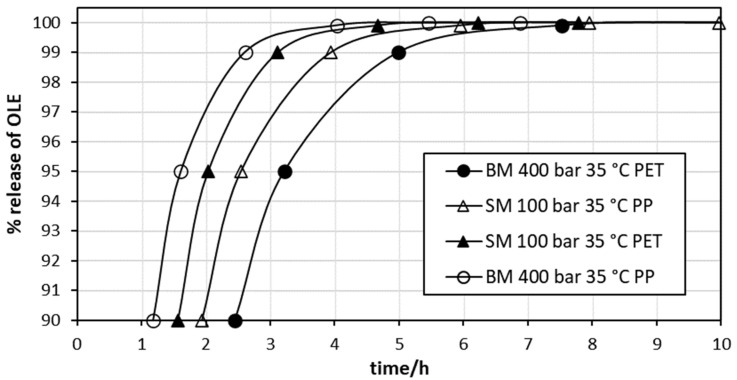
Theoretical release of OLE into food simulant.

To assess the theoretical time to obtain close to 100% release of OLE, the data obtained from Figure 4b and the linear equation coefficients presented in Table 3 were used to calculate values of *t* using selected values of *m*_t_/*m*_∞_ from Equation (8). As shown in Figure 6, close to 100% OLE was released into the simulant from all films within 8 h. Analyses such as these can be used to design films with specific release profiles. For example, an application that requires a rapid and short-term release of OLE would be suited to direct contact with the PP side of films impregnated at 400 bar using BM. Applications requiring a slower, more sustained release would be suited to direct contact with the PET side of the same film. Nevertheless, the overall film properties should be considered, and optical properties, in particular, may be a significant factor for consumers who may reject the highly coloured film obtained at the higher OLE loading.

## 4. Conclusions

Films comprised of laminated layers of PP and PET were impregnated with OLE under different conditions of pressure and impregnation mode. The OLE uptake of the resultant films modified the optical properties with significant changes in the colour imparted by the green pigmented OLE. Evaluation of the CIELAB parameters, and a* in particular, was demonstrated to efficiently correlate with the loading on impregnated PET/PP film offering a rapid and non-destructive method to quantify impregnation of coloured pigments or active agents. The UV barrier properties and water vapour permeability of the film samples were also modified but to a lesser extent. Migration of OLE into food simulants was different from the PP and PET surfaces with further differences observed at higher impregnation pressures due to the higher OLE loading achieved. The initial release rates and diffusion coefficients were higher for the PP surfaces and it was estimated that all systems reached equilibrium release within 8 h.

## Figures and Tables

**Table 1:** L*a*b* ***c***olour characteristics and OLE impregnation loading of PET/PP films prepared under different operational conditions.

Film Sample		L*	a*	b*	Δ*E* ^1^	OLE Impregnation Loading/mg g^−1^ Film ^2^
Control		94.66 ± 0.17 ^ab^	−0.47 ± 0.02 ^a^	3.43 ± 0.03 ^a^	1.02 ± 0.01 ^a^	-
BM ^3^	400 bar/35 °C	94.25 ± 0.14 ^cd^	−2.23 ± 0.01 ^f^	6.04 ± 0.04 ^ef^	3.79 ± 0.11 ^e^	2.50 ± 0.40
	100 bar/35 °C	94.63 ± 0.13 ^abc^	−1.19 ± 0.13 ^cd^	4.76 ± 0.16 ^bcd^	2.15 ± 0.14 ^d^	0.60 ± 0.25
	400 bar/55 °C	94.45 ± 0.02 ^bcd^	−1.46 ± 0.06 ^e^	4.98 ± 0.12 ^cd^	2.47 ± 0.04 ^d^	1.88 ± 0.50
	100 bar/55 °C	94.45 ± 0.18 ^d^	−1.20 ± 0.02 ^de^	4.68 ± 0.29 ^de^	2.19 ± 0.68 ^d^	0.97 ± 0.52
SM ^4^	400 bar/35 °C	94.65 ± 0.15 ^abc^	−0.85 ± 0.02 ^b^	4.12 ± 0.05 ^ab^	1.47 ± 0.04 ^ab^	1.08 ± 0.11
	100 bar/35 °C	94.05 ± 0.22 ^d^	−1.33 ± 0.19 ^e^	6.41 ± 0.21 ^f^	3.57 ± 0.08 ^e^	1.80 ± 0.03
	400 bar/55 °C	94.45 ± 0.12 ^bcd^	−1.57 ± 0.07 ^e^	4.85 ± 0.12 ^bcd^	2.04 ± 0.41 ^cd^	1.75 ± 0.01
	100 bar/55 °C	94.96 ± 0.18 ^a^	−1.05 ± 0.09 ^bc^	4.26 ± 0.29 ^bc^	1.58 ± 0.38 ^bc^	0.63 ± 0.11

Notes: different superscript letters denote significant differences among samples; ^1^ Δ*E* is the overall colour change; ^2^ data obtained from [29]; ^3^ batch mode; ^4^ semi-continuous mode.

**Table 2:** UV barrier properties of control film and films impregnated under different conditions.

Film Sample		Transparency/% mm^−1^	Opacity/mm^−1^
Control		29.36 ± 0.16 ^a^	1.04 ± 0.18 ^a^
BM ^1^	400 bar/35 °C	26.21 ± 0.44 ^b^	4.60 ± 0.69 ^b^
	100 bar/35 °C	28.77 ± 0.16 ^a^	1.70 ± 0.26 ^a^
	400 bar/55 °C	29.12 ± 0.04 ^a^	1.34 ± 0.03 ^a^
	100 bar/55 °C	28.58 ± 0.48 ^a^	1.93 ± 0.57 ^a^
SM ^2^	400 bar/35 °C	29.11 ± 0.18 ^a^	1.34 ± 0.25 ^a^
	100 bar/35 °C	29.10 ± 0.20 ^a^	1.37 ± 0.27 ^a^
	400 bar/55 °C	29.17 ± 0.05 ^a^	1.27 ± 0.06 ^a^
	100 bar/55 °C	29.01 ± 0.20 ^a^	1.44 ± 0.21 ^a^

Notes: different superscript letters denote significant differences among samples; ^1^ batch mode; ^2^ semi-continuous mode.

**Table 3:** Linear equations and correlation coefficients for first order kinetic plots.

Conditions	Surface	Linear Equation	*R* ^2^
BM ^1^ 400 bar 35 °C	PP	y = −0.027x − 0.387	0.970
	PET	y = −0.015x − 0.076	0.997
SM ^2^ 100 bar 35 °C	PP	y = −0.019x − 0.080	0.980
	PET	y = −0.025x + 0.004	0.983

Note: ^1^ batch mode; ^2^ semi-continuous mode.

**Table 4:** First-order rate constants and total mass transfer of OLE in a 50% ethanol food simulant.

Conditions	Surface	$v_{0}/\mathrm{mg}\;\min^{-1}$	*m_∞_*/mg OLE	% Release at 105 min	*D/*10^12^ m^2^ min^−1^ from Equation (10)	% Correlation between Data and Model
BM ^1^ 400 bar 35 °C	PP	0.0051	0.189	96%	8.29	99.6%
	PET	0.0031	0.204	81%	0.18	99.6%
SM ^2^ 100 bar 35 °C	PP	0.0024	0.130	87%	4.06	99.3%
	PET	0.0015	0.063	92%	0.27	99.1%

Note: ^1^ batch mode; ^2^ semi-continuous mode.

## References

[1] Amaro-Blanco G., Delgado-Adámez J., Martín M.J., Ramírez R. (2018). Active packaging using an olive leaf extract and high pressure processing for the preservation of sliced dry-cured shoulders from Iberian pigs. Innov. Food Sci. Emerg. Technol..

[2] Mir S.A., Shah M.A., Dar B.N., Wani A.A., Ganai S.A., Nishad J. (2017). Supercritical Impregnation of Active Components into Polymers for Food Packaging Applications. Food Bioprocess Technol..

[3] Bentayeb K., Rubio C., Batlle R., Nerín C. (2007). Direct determination of carnosic acid in a new active packaging based on natural extract of rosemary. Anal. Bioanal. Chem..

[4] Mousavi Khaneghah A., Hashemi S.M.B., Limbo S. (2018). Antimicrobial agents and packaging systems in antimicrobial active food packaging: An overview of approaches and interactions. Food Bioprod. Processing.

[5] Goñi M.L., Gañán N.A., Strumia M.C., Martini R.E. (2016). Eugenol-loaded LLDPE films with antioxidant activity by supercritical carbon dioxide impregnation. J. Supercrit. Fluids.

[6] Mulla M., Ahmed J., Al-Attar H., Castro-Aguirre E., Arfat Y.A., Auras R. (2017). Antimicrobial efficacy of clove essential oil infused into chemically modified LLDPE film for chicken meat packaging. Food Control..

[7] Riveros C.G., Nepote V., Grosso N.R. (2016). Thyme and basil essential oils included in edible coatings as a natural preserving method of oilseed kernels. J. Sci. Food Agric..

[8] Goñi M.L., Gañán N.A., Martini R.E., Andreatta A.E. (2018). Carvone-loaded LDPE films for active packaging: Effect of supercritical CO2-assisted impregnation on loading, mechanical and transport properties of the films. J. Supercrit. Fluids.

[9] Alirezalu K., Hesari J., Eskandari M.H., Valizadeh H., Sirousazar M. (2017). Effect of Green Tea, Stinging Nettle and Olive Leaves Extracts on the Quality and Shelf Life Stability of Frankfurter Type Sausage. J. Food Processing Preserv..

[10] Belizón M., Fernández-Ponce M.T., Casas L., Mantell C., Martínez de la Ossa-Fernández E.J. (2018). Supercritical impregnation of antioxidant mango polyphenols into a multilayer PET/PP food-grade film. J. CO_2_ Util..

[11] Cerro D., Bustos G., Villegas C., Buendia N., Truffa G., Godoy M.P., Rodriguez F., Rojas A., Galotto M.J., Constandil L. (2021). Effect of supercritical incorporation of cinnamaldehyde on physical-chemical properties, disintegration and toxicity studies of PLA/lignin nanocomposites. Int. J. Biol. Macromol..

[12] Franco P., Incarnato L., De Marco I. (2019). Supercritical CO_2_ impregnation of α-tocopherol into PET/PP films for active packaging applications. J. CO_2_ Util..

[13] Łopusiewicz Ł., Zdanowicz M., Macieja S., Kowalczyk K., Bartkowiak A. (2021). Development and Characterization of Bioactive Poly(butylene-succinate) Films Modified with Quercetin for Food Packaging Applications. Polymers.

[14] Ribeiro A.M., Estevinho B.N., Rocha F. (2020). Edible Films Prepared with Different Biopolymers, Containing Polyphenols Extracted from Elderberry (*Sambucus nigra* L.), to Protect Food Products and to Improve Food Functionality. Food Bioprocess Technol..

[15] Bouledjouidja A., Masmoudi Y., Sergent M., Trivedi V., Meniai A., Badens E. (2016). Drug loading of foldable commercial intraocular lenses using supercritical impregnation. Int. J. Pharm..

[16] Sanchez-Sanchez J., Fernández-Ponce M.T., Casas L., Mantell C., de la Ossa E.J.M. (2017). Impregnation of mango leaf extract into a polyester textile using supercritical carbon dioxide. J. Supercrit. Fluids.

[17] Dias A.M.A., Rey-Rico A., Oliveira R.A., Marceneiro S., Alvarez-Lorenzo C., Concheiro A., Júnior R.N.C., Braga M.E.M., de Sousa H.C. (2013). Wound dressings loaded with an anti-inflammatory jucá (Libidibia ferrea) extract using supercritical carbon dioxide technology. J. Supercrit. Fluids.

[18] Kazarian S.G. (2000). Polymer processing with supercritical fluids. Polym. Sci.-Ser. C.

[19] Champeau M., Thomassin J.M., Jérôme C., Tassaing T. (2014). In situ FTIR micro-spectroscopy to investigate polymeric fibers under supercritical carbon dioxide: CO_2_ sorption and swelling measurements. J. Supercrit. Fluids.

[20] Milovanovic S., Hollermann G., Errenst C., Pajnik J., Frerich S., Kroll S., Rezwan K., Ivanovic J. (2018). Supercritical CO_2_ impregnation of PLA/PCL films with natural substances for bacterial growth control in food packaging. Food Res. Int..

[21] Fanovich M.A., Ivanovic J., Misic D., Alvarez M.V., Jaeger P., Zizovic I., Eggers R. (2013). Development of polycaprolactone scaffold with antibacterial activity by an integrated supercritical extraction and impregnation process. J. Supercrit. Fluids.

[22] Rojas A., Torres A., Jose Galotto M., Guarda A., Julio R. (2020). Supercritical impregnation for food applications: A review of the effect of the operational variables on the active compound loading. Crit. Rev. Food Sci. Nutr..

[23] García-Casas I., Crampon C., Montes A., Pereyra C., Martínez de la Ossa E.J., Badens E. (2019). Supercritical CO_2_ impregnation of silica microparticles with quercetin. J. Supercrit. Fluids.

[24] Kiran E. (2016). Supercritical fluids and polymers—The year in review—2014. J. Supercrit. Fluids.

[25] Villegas C., Torres A., Bruna J., Bustos M.I., Díaz-Barrera A., Romero J., Rojas A., Guarda A. (2021). Obtaining Active Polylactide (PLA) and Polyhydroxybutyrate (PHB) Blends Based Bionanocomposites Modified with Graphene Oxide and Supercritical Carbon Dioxide (scCO_2_)-Assisted Cinnamaldehyde: Effect on Thermal-Mechanical, Disintegration and Mass Transport Properties. Polymers.

[26] Kuorwel K.K., Cran M.J., Sonneveld K., Miltz J., Bigger S.W. (2013). Migration of antimicrobial agents from starch-based films into a food simulant. LWT—Food Sci. Technol..

[27] Muppalla S.R., Kanatt S.R., Chawla S.P., Sharma A. (2014). Carboxymethyl cellulose–polyvinyl alcohol films with clove oil for active packaging of ground chicken meat. Food Packag. Shelf Life.

[28] Liu X., Jia J., Duan S., Zhou X., Xiang A., Lian Z., Ge F. (2020). Zein/MCM-41 nanocomposite film incorporated with cinnamon essential oil loaded by modified supercritical CO_2_ impregnation for long-term antibacterial packaging. Pharmaceutics.

[29] Cejudo Bastante C., Cran M.J., Casas Cardoso L., Mantell Serrano C., Martínez de la Ossa E.J., Bigger S.W. (2019). Effect of supercritical CO2 and olive leaf extract on the structural, thermal and mechanical properties of an impregnated food packaging film. J. Supercrit. Fluids.

[30] Cejudo Bastante C., Casas Cardoso L., Mantell Serrano C., Martínez de la Ossa E.J. (2017). Supercritical impregnation of food packaging films to provide antioxidant properties. J. Supercrit. Fluids.

[31] Kuorwel K.K., Cran M.J., Sonneveld K., Miltz J., Bigger S.W. (2014). Physico-mechanical properties of starch-based films containing naturally derived antimicrobial agents. Packag. Technol. Sci..

[32] Sedayu B.B., Cran M.J., Bigger S.W. (2018). Characterization of Semi-refined Carrageenan-Based Film for Primary Food Packaging Purposes. J. Polym. Environ..

[33] Colin-Chavez C., Soto-Valdez H., Peralta E., Lizardi-Mendoza J., Balandran-Quintana R. (2013). Diffusion of natural astaxanthin from polyethylene active packaging films into a fatty food simulant. Food Res. Int..

[34] Alin J., Hakkarainen M. (2010). Type of polypropylene material significantly influences the migration of antioxidants from polymer packaging to food simulants during microwave heating. J. Appl. Polym. Sci..

[35] Cejudo Bastante C., Casas Cardoso L., Fernández Ponce M.T., Mantell Serrano C., Martínez de la Ossa-Fernández E.J. (2018). Characterization of olive leaf extract polyphenols loaded by supercritical solvent impregnation into PET/PP food packaging films. J. Supercrit. Fluids.

[36] Tawakkal I.S.M.A., Cran M.J., Bigger S.W. (2016). Release of thymol from poly(lactic acid)-based antimicrobial films containing kenaf fibres as natural filler. LWT—Food Sci. Technol..

[37] Crank J. (1975). The Mathematics of Diffusion.

[38] Chalco-Sandoval W., Fabra M.J., López-Rubio A., Lagaron J.M. (2015). Development of polystyrene-based films with temperature buffering capacity for smart food packaging. J. Food Eng..

[39] Robertson G.L. (2009). Food Packaging and Shelf Life: A Practical Guide.

[40] Wrona M., Cran M.J., Nerín C., Bigger S.W. (2017). Development and characterisation of HPMC films containing PLA nanoparticles loaded with green tea extract for food packaging applications. Carbohydr. Polym..

[41] Wang L.-F., Rhim J.-W. (2016). Grapefruit seed extract incorporated antimicrobial LDPE and PLA films: Effect of type of polymer matrix. LWT.

[42] Kanmani P., Rhim J.-W. (2014). Antimicrobial and physical-mechanical properties of agar-based films incorporated with grapefruit seed extract. Carbohydr. Polym..

[43] Burkinshaw S.M. (2015). Physico-Chemical Aspects of Textile Coloration.

[44] Varga D., Alkin S., Gluschitz P., Péter-Szabó B., Székely E., Gamse T. (2016). Supercritical fluid dyeing of polycarbonate in carbon dioxide. J. Supercrit. Fluids.

[45] Abate M.T., Ferri A., Guan J., Chen G., Ferreira J.A., Nierstrasz V. (2019). Single-step disperse dyeing and antimicrobial functionalization of polyester fabric with chitosan and derivative in supercritical carbon dioxide. J. Supercrit. Fluids.

[46] Solovieva A.B., Cherkasova A.V., Glagolev N.N., Kopylov A.S., Timashev P.S., Tsypina S.I., Bagratashvili V.N. (2017). Stable “coloured” states of spirooxazine photochrom molecules immobilized in polymer matrixes by supercritical carbon dioxide. J. Mol. Liq..

[47] Champeau M., Thomassin J.M., Tassaing T., Jerome C. (2015). Drug Loading of Sutures by Supercritical CO_2_ Impregnation: Effect of Polymer/Drug Interactions and Thermal Transitions. Macromol. Mater. Eng..

[48] Goñi M.L., Gañán N.A., Herrera J.M., Strumia M.C., Andreatta A.E., Martini R.E. (2017). Supercritical CO_2_ iof LDPE films with terpene ketones as biopesticides against corn weevil (*Sitophilus zeamais*). J. Supercrit. Fluids.

[49] Dias A.M.A., Braga M.E.M., Seabra I.J., Ferreira P., Gil M.H., de Sousa H.C. (2011). Development of natural-based wound dressings impregnated with bioactive compounds and using supercritical carbon dioxide. Int. J. Pharm..

[50] Rojas A., Cerro D., Torres A., Galotto M.J., Guarda A., Romero J. (2015). Supercritical impregnation and kinetic release of 2-nonanone in LLDPE films used for active food packaging. J. Supercrit. Fluids.

[51] Alvarado N., Romero J., Torres A., López de Dicastillo C., Rojas A., Galotto M.J., Guarda A. (2018). Supercritical impregnation of thymol in poly(lactic acid) filled with electrospun poly(vinyl alcohol)-cellulose nanocrystals nanofibers: Development an active food packaging material. J. Food Eng..

[52] Shankar S., Wang L.-F., Rhim J.-W. (2018). Incorporation of zinc oxide nanoparticles improved the mechanical, water vapor barrier, UV-light barrier, and antibacterial properties of PLA-based nanocomposite films. Mater. Sci. Eng. C.

[53] Wu C., Sun J., Zheng P., Kang X., Chen M., Li Y., Ge Y., Hu Y., Pang J. (2019). Preparation of an intelligent film based on chitosan/oxidized chitin nanocrystals incorporating black rice bran anthocyanins for seafood spoilage monitoring. Carbohydr. Polym..

[54] Hashmi S. (2014). Comprehensive Materials Processing.

[55] Sangaj N.S., Malshe V.C. (2004). Permeability of polymers in protective organic coatings. Prog. Org. Coat..

[56] Siripatrawan U., Vitchayakitti W. (2016). Improving functional properties of chitosan films as active food packaging by incorporating with propolis. Food Hydrocoll..

[57] Sanchez-Garcia M.D., Lagaron J.M. (2010). Novel clay-based nanobiocomposites of biopolyesters with synergistic barrier to UV light, gas, and vapour. J. Appl. Polym. Sci..

[58] Benbettaïeb N., Tanner C., Cayot P., Karbowiak T., Debeaufort F. (2018). Impact of functional properties and release kinetics on antioxidant activity of biopolymer active films and coatings. Food Chem..

[59] Champeau M., Thomassin J.M., Tassaing T., Jérôme C. (2015). Drug loading of polymer implants by supercritical CO_2_ assisted impregnation: A review. J. Control. Release.

[60] Marcos B., Sárraga C., Castellari M., Kappen F., Schennink G., Arnau J. (2014). Development of biodegradable films with antioxidant properties based on polyesters containing α-tocopherol and olive leaf extract for food packaging applications. Food Packag. Shelf Life.

[61] Torres A., Romero J., Macan A., Guarda A., Galotto M.J. (2014). Near critical and supercritical impregnation and kinetic release of thymol in LLDPE films used for food packaging. J. Supercrit. Fluids.

[62] Rojas A., Torres A., Añazco A., Villegas C., Galotto M.J., Guarda A., Romero J. (2018). Effect of pressure and time on scCO_2_-assisted incorporation of thymol into LDPE-based nanocomposites for active food packaging. J. CO2 Util..

